# Microfluidic preparation of anchored cell membrane sheets for *in vitro* analyses and manipulation of the cytoplasmic face

**DOI:** 10.1038/s41598-017-14737-7

**Published:** 2017-11-02

**Authors:** Shin Izuta, Satoshi Yamaguchi, Ryuji Misawa, Shinya Yamahira, Modong Tan, Masahiro Kawahara, Tomoko Suzuki, Tomoko Takagi, Kae Sato, Motonao Nakamura, Teruyuki Nagamune, Akimitsu Okamoto

**Affiliations:** 1 Department of Chemistry and Biotechnology, Graduate School of Engineering, The University of Tokyo, 7-3-1 Hongo, Bunkyo-ku, Tokyo, 113-8656 Japan; 2 Research Center for Advanced Science and Technology, The University of Tokyo, 4-6-1 Komaba, Meguro-ku, Tokyo, 153-8904 Japan; 3 PRESTO, Japan Science and Technology Agency (JST), Tokyo, Japan; 4 Department of Chemical and Biological Sciences, Japan Women’s University, 2-8-1 Mejirodai, Bunkyo-ku, Tokyo, 112-8681 Japan; 5 Department of Life Science, Faculty of Science, Okayama University of Science, 1-1 Ridai-cho, Kita-ku, Okayama-shi, Okayama, 700-0005 Japan

## Abstract

Molecular networks on the cytoplasmic faces of cellular plasma membranes are critical research topics in biological sciences and medicinal chemistry. However, the selective permeability of the cell membrane restricts the researchers from accessing to the intact intracellular factors on the membrane from the outside. Here, a microfluidic method to prepare cell membrane sheets was developed as a promising tool for direct examination of the cytoplasmic faces of cell membranes. Mammalian cells immobilized on a poly(ethylene glycol)-lipid coated substrate were rapidly and efficiently fractured, with the sheer stress of laminar flow in microchannels, resulting in isolation of the bottom cell membrane sheets with exposed intact cytoplasmic faces. On these faces of the cell membrane sheets, both ligand-induced phosphorylation of receptor tyrosine kinases and selective enzymatic modification of a G-protein coupling receptor were directly observed. Thus, the present cell membrane sheet should serve as a unique platform for studies providing new insights into juxta-membrane molecular networks and drug discovery.

## Introduction

**Figure 1:**
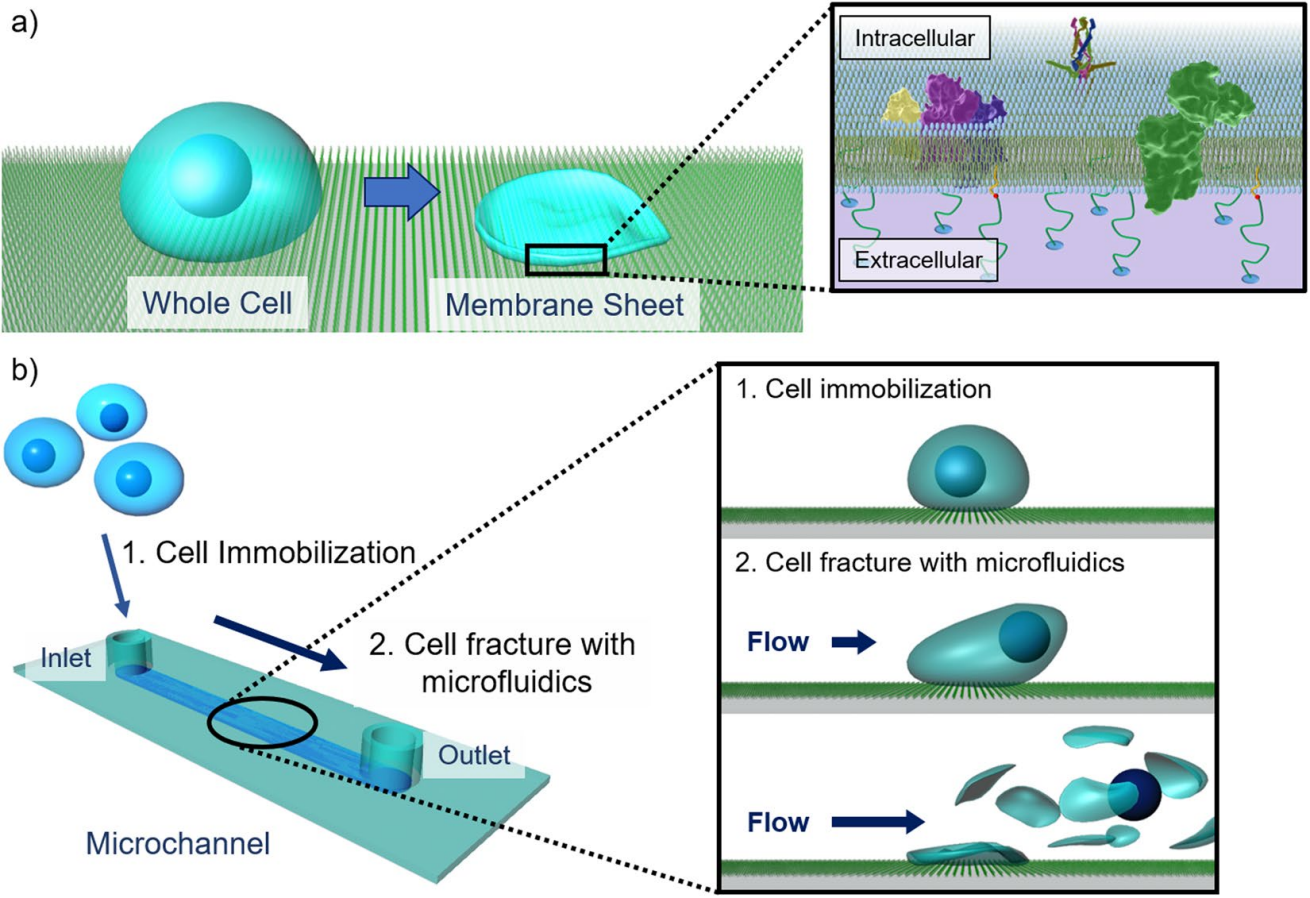
Schematic illustrations of cell membrane sheets and the microfluidic device. (**a**) Schematic illustrations of cell membrane sheets on glass substrate and (**b**) preparation of cell membrane sheets from living cells with a microfluidic device.

Molecular networks around the cell membrane are critical interfaces between the extracellular environments and the intracellular living systems and have been actively studied in a variety of research fields, from fundamental molecular biology to drug discovery^1,2^. Networks at the extracellular face are widely investigated with conventional molecular tools, such as antibodies and agonists/antagonists^3,4^. However, there are very few methods for examining the cytoplasmic face, because the selective permeability of the cell membrane restricts access to intact intracellular factors from the outside. Therefore, conventional techniques for molecular imaging and drug screening cannot be applied to intracellular juxtamembrane factors without damaging membrane structures^5^. Genetic engineering techniques can be employed for controlling and visualizing molecules on the intact cytoplasmic face^6^. However, there are limitations: (1) the complicated and unreliable gene expression processes, from DNA to active proteins are included; (2) synthetic chemicals and chemically functionalized biomolecules would be either unavailable or difficult to use; and (3) fusion to marker fluorescent proteins might disturb properties of the original proteins, because of steric bulk or electrostatic charges^7,8^. Thus, there is an unmet need for simple methods to investigate the intact cytoplasmic face for chemical biology, pharmaceutical and medicinal chemistry studies. Here, we developed a new method to obtain intact cell membrane sheets from living cells, enabling direct assessment of the intact cytoplasmic face (Fig. 1a). In this method, cells were attached to substrates and their plasma membranes were then fractured to remove both the top plasma membranes and cytoplasmic organelles. This left a remaining bottom membrane sheet, with the intact cytoplasmic face fully exposed. This cytoplasmic face could be treated directly with a variety of molecular probes and enzymes, as well as analyzed using various imaging methods.

For the past twenty years, inside-out cell membrane fragments were employed to investigate the cytoplasmic face of chemically fixed membranes, by electron microscopy^9^ and atomic force microscopy^10^. This approach was recently also applied to fluorescence imaging of molecules on cell membranes^11,12^. In these previous studies, to prepare membrane fragments, cells were attached to positively charged surfaces through electrostatic interactions and various methods such as hypotonic solutions^6,13^, sonication^10^ and peeling^12^ were used to fracture the plasma membranes. However, such electrostatic interactions were reported to cause cytotoxicity^14^. Conventional hypotonic solution treatment damaged membrane structure through osmotic swelling, leading to cell disruption only by gentle rinsing^13^. In addition, sonication, a commonly used cell fracturing method^10^, did not prepare uniform membrane sheets with highly reproducible shapes, sizes, states of the membrane surface or degree of fracture because it caused unevenly variable shear stress at each position and each trial. Therefore, these conventionally prepared cell membrane fragments are not suited for accurate investigation of biological events on the intact cytoplasmic face.

Our strategy for rapidly preparing intact cell membrane sheets is as follows (Fig. 1b): (1) the bottom glass surfaces of microchannels were coated by lipids with a poly(ethylene glycol) (PEG) linker; (2) cells were immobilized on these surfaces through interactions between the lipid moieties and cell membranes^15,16^; (3) the immobilized cells were fractured using laminar microchannel flow, resulting in preparation of intact cell membrane sheets. In this method, the shear stress of laminar flow was applied to the cells in parallel with the substrate, such that the bottom membrane received no direct stress. The PEG–lipid used in our study was reported to immobilize cells without causing cytotoxicity^15,16^. Additional potential advantages of this microfluidic system are that cell fracture can be performed with real-time microscopic observation and that it should require only small amounts of costly reagents for molecular analyses after cell fracture.

## Results

### Preparation and validation of cell membrane sheets

**Figure 2:**
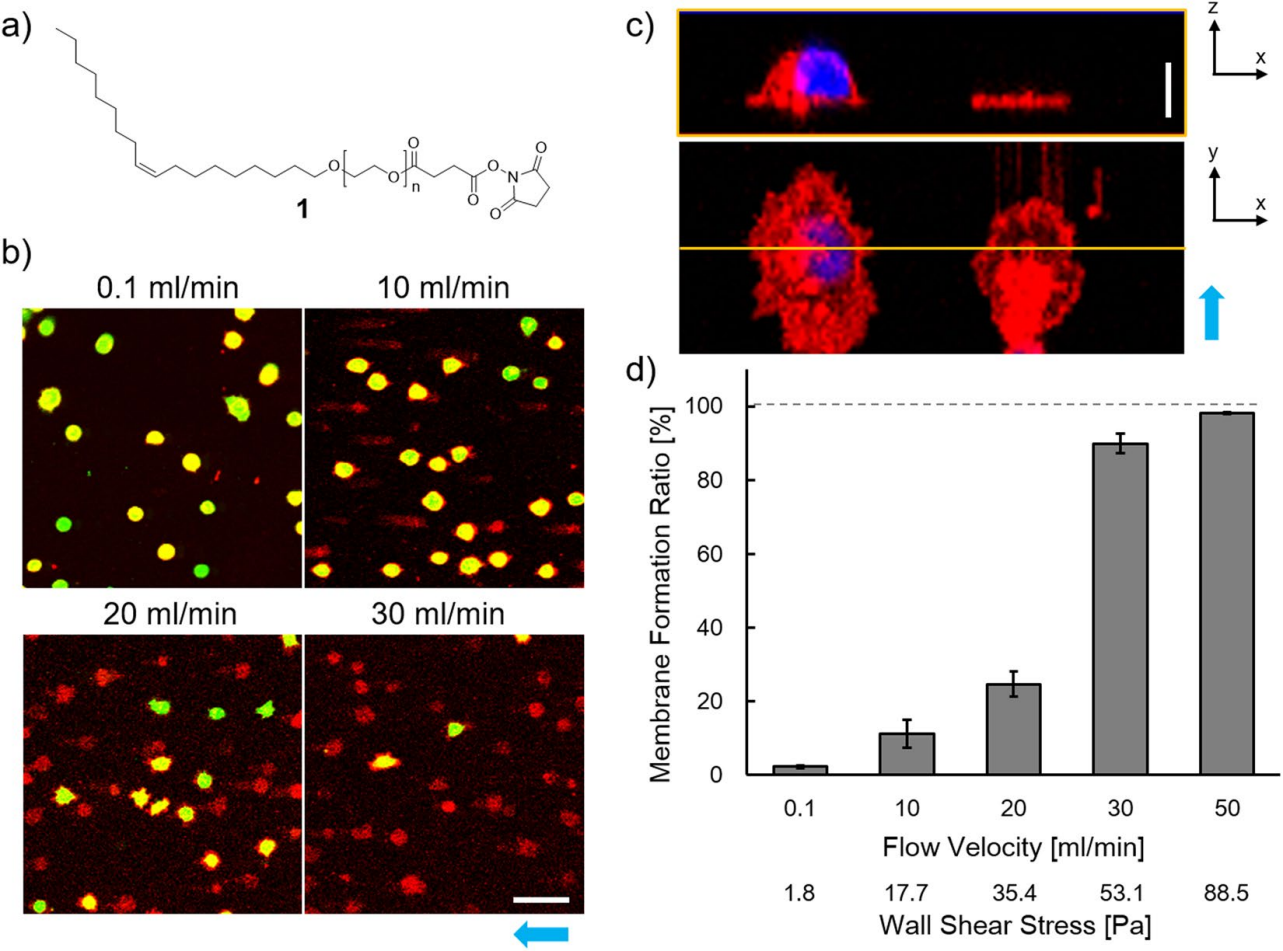
Confocal microscopy images of cell membrane sheets. (**a**) Chemical structure of lipidation reagent 1, based on PEG–lipid. (**b**) Confocal fluorescence images of immobilized whole cells and membrane sheets after treatment with laminar flow at various flow velocities. The cytoplasm and plasma membrane of Ba/F3 cells were stained with CalceinAM (green) and Alexa Fluor 647 conjugated PEG–lipid (red), respectively. The stained cells were immobilized and exposed to flow for 1 min at linear flow velocities from 0.1 to 30 mL/min. Scale bar, 50 μm. (**c**) Cross sections of confocal 3D reconstruction images of immobilized whole cells (*left*) and membrane sheets after treatment with laminar flow at 50 mL/min (*right*). The (top) x-z and (bottom) x-y sections were obtained from the 3D image, which was reconstructed by composing a series of 2D z-stack confocal images of cells stained with DiI (plasma membrane, red) and Hoechst33342 (nucleus, blue). The x–z section image was made at the yellow line in the x-y section image. Scale bars, 10 μm. (**d**) The ratio of the cell membrane sheet formation at each flow velocity. Values are means ± standard error (n = 27, from three independent trials). Blue arrows show the direction of flow for cell fracture.

Double stained Ba/F3 cells (a murine pro-B cell line), with cytoplasm and plasma membrane fluorescently stained with CalceinAM and Alexa Fluor 647 (AF647)-labeled PEG–lipid (Supplementary Fig. S1), respectively, were immobilized on the lipid modified surface. This surface had been prepared on collagen coated glass slides with lipidation reagent **1** (Fig. 2a). A physiological buffer was poured into the microchannel, at various linear flow velocities, to fracture the immobilized cells. After exposure to the flow for 1 min at a low flow velocity, most of the cells remained with both Calcein (green) and AF647 fluorescence (red) (Fig. 2b, 0.1 mL/min). At a flow velocity of 10 mL/min, several red structures without green fluorescence were observed on the substrate and their numbers were increased at higher flow velocities (Fig. 2b and d). This indicated that the cytoplasm was removed by cell fracture in response to the shear stress of laminar flow. The vertical (x-z) section of the 3D reconstruction images showed complete removal of the top plasma membranes and the nuclei, with only thin red-stained membrane remaining (Fig. 2c), believed to be the bottom membrane of the fractured cells. Similar fluorescence images were obtained in equivalent experiments using human cervical carcinoma (He/La) cells (Supplementary Fig. S3). Therefore, this method can be applied to both adherent and nonadherent cells, although the efficiency of the membrane sheet formation from well-spread adherent cells drastically decreased after long incubation.

**Figure 3:**
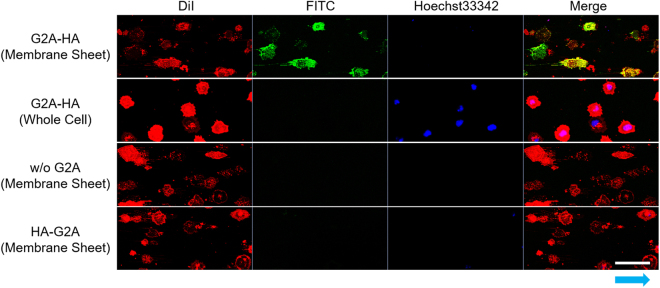
Confocal microscopy images of immunostained cell membrane sheets. Confocal fluorescence images of Ba/F3 cells expressing G2A with HA-tag sequence at the extracellular N-terminus (HA–G2A) and the intracellular C-terminus (G2A–HA). Whole cells or membrane sheets were stained with anti–HA antibody FITC conjugate (for HA-tag, green), DiI (for plasma membrane, red) and Hoechst33342 (for nucleus, blue). Scale bar, 50 μm. Blue arrow shows the direction of flow for cell fracture.

Exposure of the cytoplasmic face of plasma membranes was confirmed by immunostaining of a tagged membrane protein, before and after microfluidic cell fracture. We selected the G-protein coupled receptor (GPCR) G2A as the membrane protein and fused a HA-tag at either the extracellular N-terminus or the intracellular C-terminus of G2A. These fused products were designated as HA–G2A and G2A–HA, respectively. The cell membrane sheets were prepared from Ba/F3 cells expressing tagged G2A, at a flow velocity of 50 mL/min, and were stained with a fluorescently labeled anti-HA antibody. Immunofluorescence was observed only in membrane sheets from cells expressing G2A–HA (Fig. 3). This membrane sheet was anchored above the glass substrate through the PEG layer, and the added antibody did not intrude into the layer between the cell membrane and the substrate (Supplementary Fig. S4). This immunostaining assay showed that, after microfluidic fracture of the cells, the cytoplasmic face of the membrane was exposed to the solution.

### Surface and fluidic characteristics of cell membrane sheets

**Figure 4:**
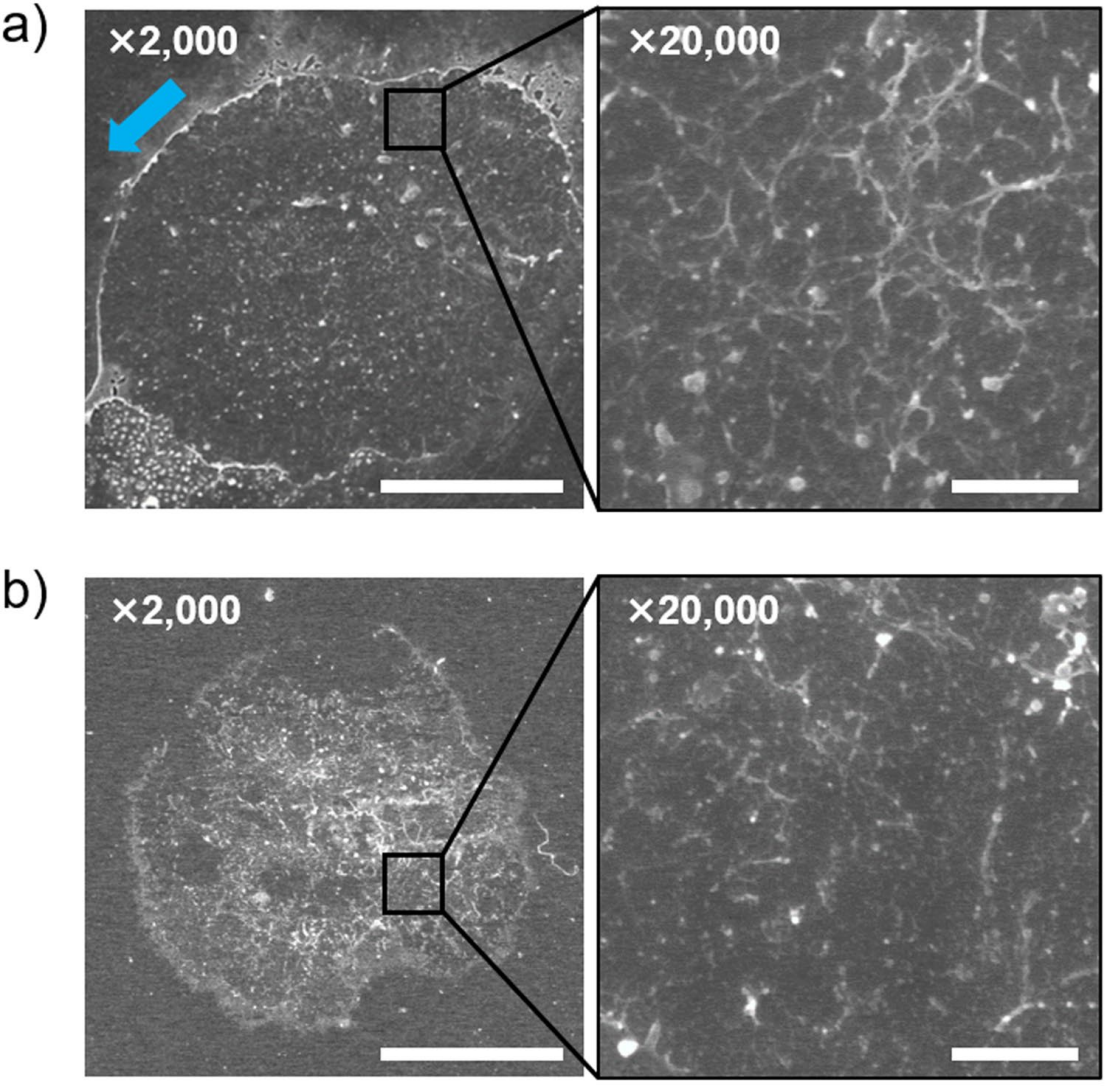
Scanning electron microscopy images of cell membrane sheets. Scanning electron microscopy images of a membrane sheet prepared from Ba/F3 cells with (**a**) the microfluidic method and (**b**) the sonication method. Scale bar, 10 μm (2000×, left), 1 μm (20,000×, right). Blue arrow shows the direction of flow for cell fracture.

The surface of this membrane sheet was also analyzed by scanning electron microscopy (SEM). The SEM image showed that the cell membrane sheet had a uniform surface, with dispersed nanostructures (Fig. 4a). The laminar flow used to prepare the sheet shown in Fig. 4a was applied from the upper left toward the lower right. The membrane edge at the upper side of the image was slightly turned up and the edge at the lower side appeared to have been pulled outward (Fig. 4a and Supplementary Fig. S5). This SEM image suggested that the laminar flow sharply cut the side plasma membranes, in principle without significant damage to the bottom membranes. In high-magnification images, both fibrous and particulate structures, with submicrometer sizes, were observed on the membrane surface (Fig. 4a, right image). Fibrous protein networks, consisting of spectrin and actin, which support the plasma membrane, were reportedly observed by SEM on fixed erythrocyte membranes^17^. From their sizes and shapes, the fibrous structures in Fig. 4a appeared to correspond to such networks.

**Figure 5:**
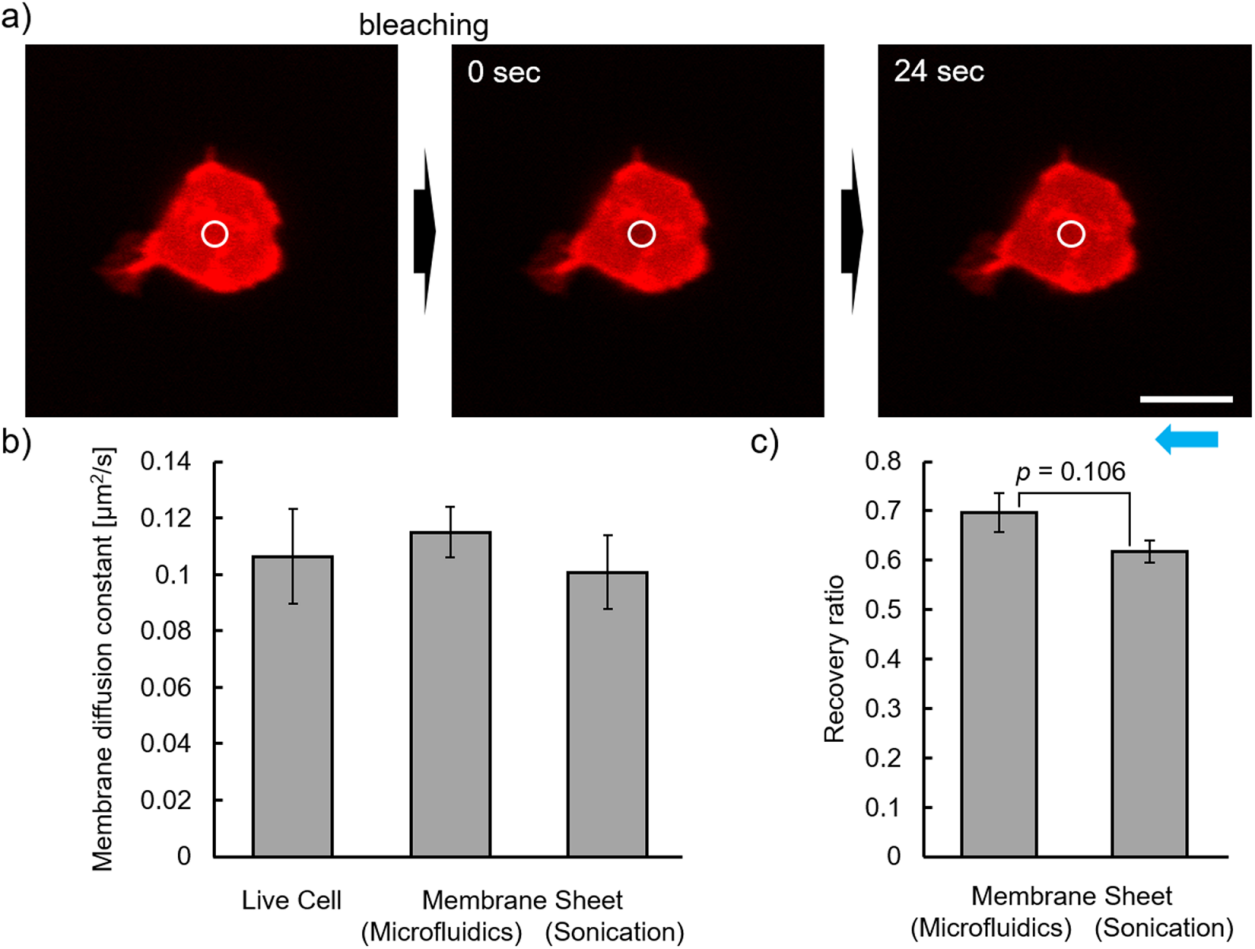
Fluorescence recovery after photo-bleaching (FRAP) analysis of cell membrane sheets. FRAP analysis data for Ba/F3 cells were obtained on whole cells and the membrane sheet. (**a**) Confocal microscopic images of the cell membrane sheets in the FRAP experiment. The red fluorescent images of the DiI-stained membrane sheet was obtained before (*left*), just after (*center*) and 24 sec after bleaching (*right*). The white circle shows the breached region. Scale bar, 10 μm. (**b**) The lateral diffusion coefficients and (**c**) the recovery ratios of the fluorescence intensity at the bleached areas were shown. Values are means ± standard error (n = 10). The *p*–values were calculated with Student’s *t*–test. Blue arrow shows the direction of flow for cell fracture.

Fluidity of the membrane sheets was examined using fluorescence recovery after photobleaching (FRAP)^18^. The rate of lateral motion of DiI, a lipid-like fluorescent dye, was measured on the bottom membranes in both whole live cells and cell membrane sheets (Fig. 5a). Figure 5b shows the lateral diffusion coefficients of DiI for each membrane. There was no significant difference between the coefficients of the live cells and the membrane sheets, suggesting that the cell fracture procedure had negligible effects on membrane fluidity. There were also no significant differences in diffusion coefficients between samples prepared by microfluidic and sonication methods (Fig. 5b). In contrast, by FRAP, the recovery ratio tended to be slightly lower for the sonication than for the microfluidic method, although it was not statistically significant (Fig. 5c). The lower fluorescence recovery observed in samples prepared by sonication was presumably derived from fragmentation of the membrane sheet. Thus, cell membrane sheets with characteristics similar to those of intact plasma membranes were readily obtained by our microfluidic method.

### Phosphorylation detection on cell membrane sheets

**Figure 6:**
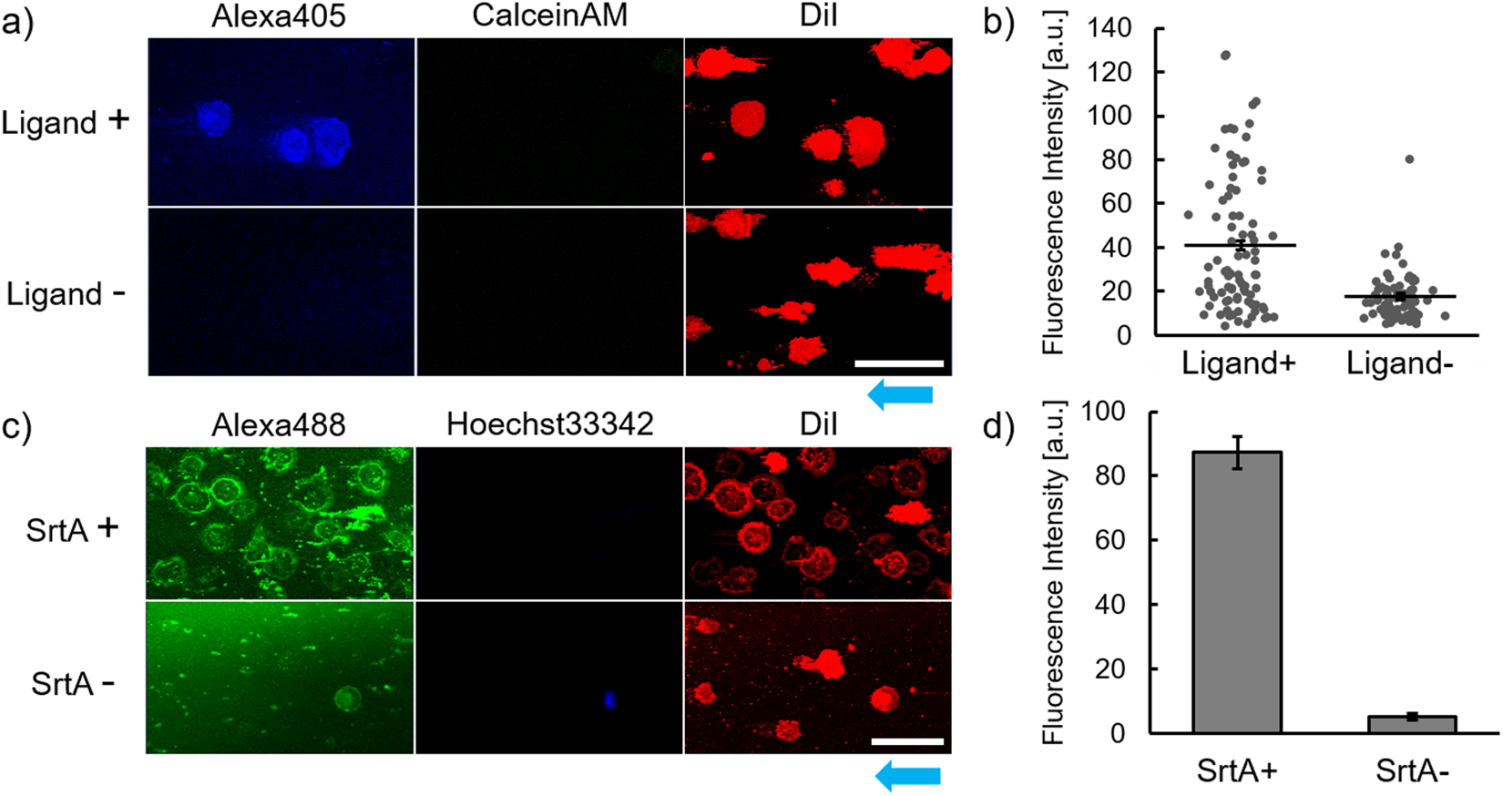
Confocal microscopy for ligand-induced phosphorylation and enzymatic modification of membrane receptors on cell membrane sheets. (**a**) Images of membrane sheets from Ba/F3 cells, treated with and without the ligand. The membrane sheets of cells expressing receptor tyrosine kinase were stained with anti-p-Tyr antibody Alexa405 conjugate (for p-Tyr, blue), Calcein AM (for cytoplasm, green) and DiI (for plasma membrane, red). (**b**) Fluorescent intensities of the labeled anti-p-Tyr antibody on the individual cell membrane sheet. The intensities on each membrane sheet of the cell treated with and without the ligand were plotted (ligand+: n = 89, ligand−: n = 72). The black bars and the error bars show the mean intensity and the standard error, respectively (*p* < 0.001 in Student’s *t-*test). (**c**) Images of membrane sheets, treated with and without sortase A, in a solution of Alexa Fluor 488 (Alexa488)-labeled substrate peptide. Cells were stained with DiI (for plasma membrane, red) and Hoechst33342 (for nucleus, blue). Scale bar, 50 μm. (**d**) Fluorescent intensities of the labeling reagent on the cell membrane sheets. The intensities on each membrane sheet of the cell treated with and without sortase A (SrtA) were obtained (SrtA+: n = 64, ligand−: n = 33). The values represent the mean intensity and the standard error, respectively (*p* < 0.001 in Student’s *t-*test). Blue arrows show the direction of flow for cell fracture.

The cell membrane sheets generated by microfluidics should be useful for detecting molecular events on the cytoplasmic face. To demonstrate this, we focused on ligand-induced phosphorylation of receptor tyrosine kinases (RTKs), which activate signaling pathways controlling cell proliferation and differentiation^19^. We prepared Ba/F3 cells expressing an artificial chimeric receptor^20^, which could activate the JAK–STAT pathway by phosphorylation, in response to a specific bio-orthogonal ligand. These cells were treated with the ligand and then immobilized and fractured in a microchannel. Tyrosine phosphorylation in the cytoplasmic domain of the receptor was then stained with a fluorescently labeled anti-p-Tyr antibody. This fluorescent staining was evident in the cell membrane sheet (Fig. 6a, ligand + ), with negligible staining observed on a sheet prepared from the cells not treated with the ligand (Fig. 6a, ligand −). Phosphorylation at the cytoplasmic domain of the receptor was, thus, easily visualized in cell membrane sheets. In the resulting images, ligand-induced activation was detected on almost half of the cells (Fig. 6a and b). Such information about receptor state, at a single cell level, is important for gaining new insights into diversity among individual cells, not possible with conventional methods of bulk analysis. Furthermore, with this microfluidic method, cells can be rapidly immobilized, fractured and then treated with analytical reagents soon after cell fracture. Immunostaining can be started within 15 min after receptor activation and fixation is unnecessary for accessing the intracellular domain of the receptor with probes. These advantages would potentially expand the range of observable molecular events on the cytoplasmic face of membranes.

### Enzymatic reaction on cell membrane sheets

The *in vitro* approaches using reconstruction of molecular systems in a test tube has been actively challenged to quantitatively describe relationships among various factors in biological processes^21,22^. Recently, *in vitro* reconstruction of the juxtamembrane system was attempted^23–26^, however, naturally-occuring molecular systems on the plasma membrane are too sophisticated and complex to be fully reconstructed by incorporating isolated synthetic components. Our cell membrane sheet, containing whole membrane components with their intrinsic orientations and densities preserved, could serve as a platform for bottom-up reconstruction of the intracellular juxtamembrane system. We examined enzymatic modification of one membrane protein on the cytoplasmic face, as a model biological event. To confirm the designed modification, the tagged intracellular domain of a model GPCR was ligated with a fluorescently labeled peptide, using Sortase A (SrtA)^27^. The fluorescent peptide was clearly observed on all membrane sheets after SrtA-mediated transpeptidation (Fig. 6c). Meanwhile, on the surface treated with the fluorescent peptide alone, there was little fluorescent staining on the membrane sheets (Fig. 6d), and only nonspecific adsorption of the fluorescent peptide was observed in the whole cells that had failed to be fractured. Thus, we demonstrated that, with these cell membrane sheets, a membrane protein of interest can be selectively modified through an enzymatically reaction.

### Photo-guided array of cell membrane sheets

**Figure 7:**
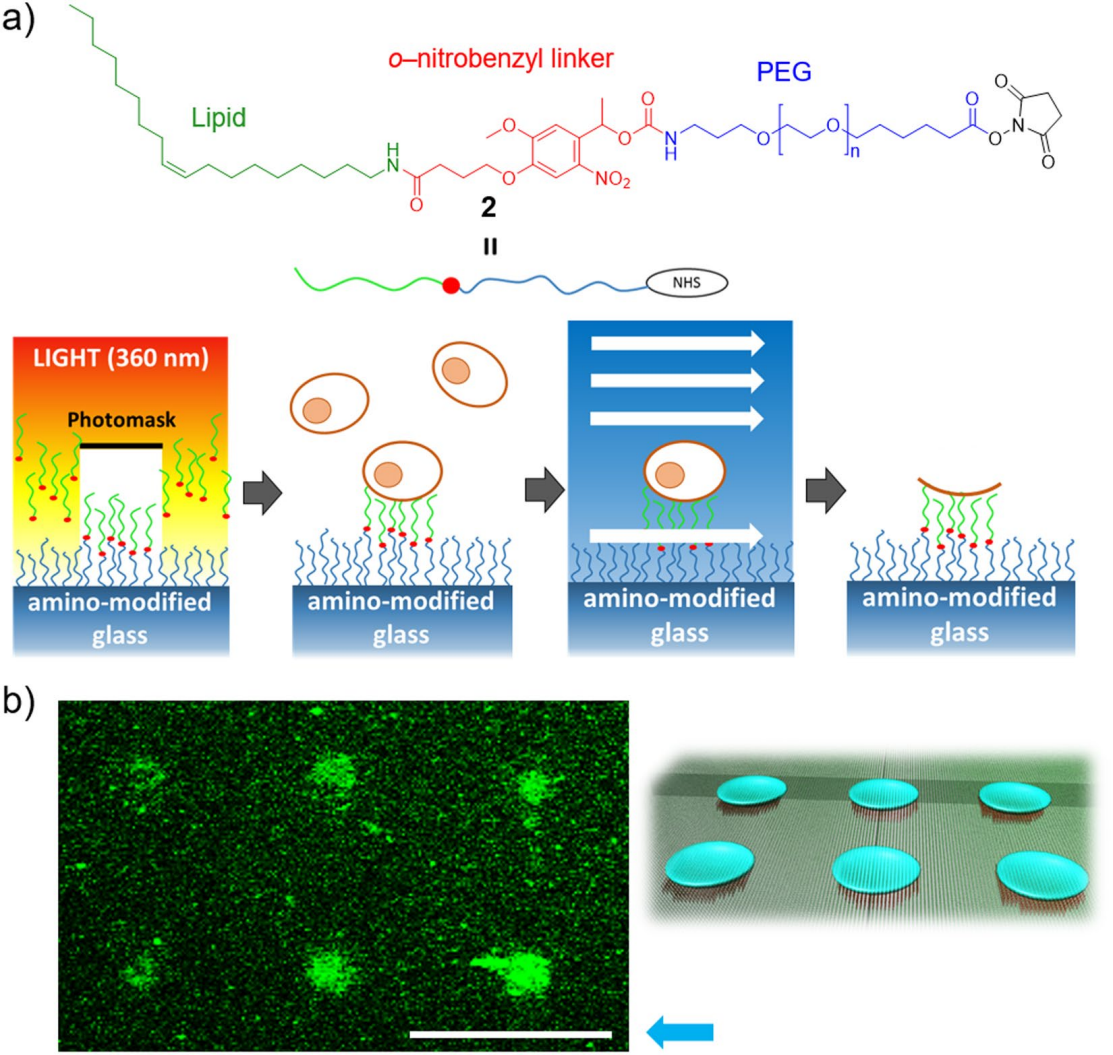
Light-guided array of cell membrane sheets. (**a**) Chemical structure of lipidation reagent 2, based on a photocleavable PEG–lipid, and schematic illustration of light-guided patterning of the cell membrane sheets. (**b**) Confocal microscopic image of a light-guided array of membrane sheets at the single-cell level (*left*) and illustration of the membrane sheet array (*right*). The membrane sheets were stained with fluorescein-conjugated PEG–lipid. Scale bar, 50 μm. Blue arrow shows the direction of flow for cell fracture.

Quantitative and exhaustive single-cell analyses can provide new and accurate insights that would be masked by the intrinsic heterogeneity of a cell population^28^. Use of the photo-responsive lipidation reagent **2** ^29,30^ enabled construction of an array of cell membrane sheets, at a single cell level (Fig. 7a). In light irradiated areas, lipid moieties could be removed through light-induced cleavage of the *o*–nitrobenzyl linker, exposing PEG surfaces not adhered to cells. Cells were, thus, selectively immobilized only at the non-irradiated areas, enabling formation of an array of cell membrane sheets using our microfluidic method (Fig. 7b). Such cell membrane sheet arrays could potentially enable high throughput analysis of the membrane sheets by positioning them in a high density reticular pattern. In such a light-guided system, the size and shape of the membrane sheet could potentially be controlled by the pattern of light, to achieve more accurate quantitative analysis.

## Discussions

In the current study, we developed a simple microfluidic method to prepare cell membrane sheets with their intact cytoplasmic faces exposed. Our method reliably transformed most cells on PEG–lipid coated surfaces into cell membrane sheets with a uniform surface, using microfluidic shear stress-induced cell fracture. In contrast, the surface of the cell membrane sheet prepared by conventional sonication was uneven, probably because cavitation bubbles were randomly formed and collapsed both the membranes and intracellular structures (Fig. 4b, *left*). Most of these surfaces were non-uniformly covered with remaining intracellular structures and the membrane faces appeared only in patches. The enlarged view showed that the fibers supporting the membrane were fragmented (Fig. 4b, *right*). Thus, compared with the sonication method, our microfluidic method was suggested to produce more even plasma membrane sheets, with uniformly exposed membrane faces and intact intracellular cortical structures (Fig. 4a). In the present system, cells are instantly fractured within a few seconds at 50 mL/min. Accordingly, shear stress-induced biological events such as cytoskeletal remodeling (from tens of minutes to hours) are negligible^31,32^. On the cell membrane sheets, we detected ligand-induced phosphorylation of RTKs by immunostaining on the cytoplasmic face. Such phosphorylation of RTKs is the key trigger of the intracellular signaling pathways, and a variety of signal transduction factors are recruited to those phosphorylated domain. Therefore, these cell membrane sheets could be a promising tool for examining recruitment of intracellular signal transduction factors on activated receptors and for screening their inhibitors. The cell membranes are potentially prepared on a variety of sensor surfaces because the present method needs only sheer stress. For example, they may be applied to surface plasmon resonance (SPR) sensor for kinetic analysis of the interaction between phosphorylated receptors and signal transduction factors. In future, it is better to allow access to both sides of the membrane sheets. Recently, molecular devices which can spatio-temporally release ligands from the substrate surface were reported to load the ligands to the cells above the device-modified substrate^33^. Combined use of the present cell membrane sheet and such a ligand-release system is expected to achieve *in situ* loading of ligands from the underside of the sheet to the receptor and enable real-time observation and modulation of more rapid signal transduction on the cytoplasmic face. Moreover, the intracellular domain of a model GPCR was selectively modified enzymatically. In this study, to simply detect the modification of the intracellular domain, the tagged GPCR was modified with a fluorescent dye. Similarly, intact endogenous molecules on the cytoplasmic face could potentially be modified by various enzymes, such as kinases and ubiquitin ligase, to identify those responsible enzymes involved in juxtamembrane post-translational modifications (PTMs) related to diseases and other important biological phenomena. In conventional methods using a ‘knock-in and knock-out’ strategy, it is not simple to clarify a causal relation between those PTMs and responsible enzymes in the complex molecular systems. On the present membrane sheet, purified enzymes, substrates and other related factors can be accessed to membrane proteins in a designed manner. Furthermore, synthetic chemicals and chemically modified biomolecules should also be able to interact with cell membrane sheets on the cytoplasmic side. Using our approach, investigators can readily evaluate the functions of both natural and synthesized molecules on the cytoplasmic face, enabling systematic studies of the molecular processes occurring in intracellular juxtamembrane regions. As discussed above, the present microfluidic method makes us conceive a variety of possible applications for bioscience and pharmaceutical researches. This is because the uniformly exposed intact cytoplasmic faces can be treated with high reproducibility and reliability just after cell fracture, different from other conventional methods. Finally, the photo-guided manipulation system for the cell membrane sheets was briefly demonstrated by using our photocleavable lipidation reagent. On the photo-responsive surface, prepared cell membrane sheets are potentially detached by light-induced release of the anchoring lipid moiety from the substrate for further analysis and use of the membrane molecules. Thus, these cell membrane sheets should serve as a unique platform for studies providing new insights into molecular networks on the cytoplasmic face of the cell membrane.

## Methods

### Materials

Unless otherwise specified, the chemicals used in the current work were of analytical grade and used without further purification. 1,1′-Dioctadecyl-3,3,3′,3′-tetramethylindocarbocyanine perchlorate (DiI), Alexa Fluor 647-*N*-hydroxy succinimidyl ester (Alexa647–NHS) and Alexa Fluor 488-maleimide were from Molecular Probes (Eugene, OR, USA) and Hoechst33342, CalceinAM and FITC (fluoroscein-4-isothiocyanate) were from Dojindo Laboratories (Kumamoto, Japan). Lipidation reagent **1** (Sunbright OE-040CS), bifunctionalized poly(ethylene glycol)(PEG) (Boc-*N*-amine-PEG–NHS, Sunbright BO-050TS) and 1,2-dioleoyl-sn-glycero-3-phosphoethanolamine (DOPE) were from NOF Corporation (Tokyo, Japan). RPMI-1640 medium was from Wako Pure Chemicals (Osaka, Japan). Fetal bovine serum was from Thermo Fisher Scientific (Waltham, MA). Murine interleukin (IL)-3 and Dulbecco’s modified Eagle’s medium were from Gibco BRL (Gaithersburg, MD). Puromycin, bovine serum albumin (BSA) and BSA fluorescein conjugate (FL–BSA) was from Sigma Aldrich (St. Louis, MO). Penicillin/streptomycin was from Nacalai Tesque (Kyoto, Japan). Collagen was from Nitta Gelatin Inc. (Osaka, Japan). Anti–HA rabbit polyclonal antibody FITC conjugate was from Bethyl Laboratories Inc. (Montgomery, TX). Anti-p-Tyr antibody AlexaFluor405 conjugate was from Santa Cruz Biotechnology, Inc. (Dallas, TX). Glutaraldehyde for electron microscopy was from Electron Microscopy Sciences (Fort Washington, PA). Fluorescently labeled PEG–lipids were synthesized essentially as previously reported^16^. (Supplementary Fig. S1).

### Cell preparation

A murine IL-3-dependent pro-B cell line, Ba/F3 and a human epithelial carcinoma cell line, HeLa, were from RIKEN BioResource Center (BRC, Tsukuba, Japan). Ba/F3 cells expressing tagged G2As were prepared essentially as previously described^34^. Expression vectors were prepared encoding the tagged G2As, on which a HA-tag sequence and a LPETGGGGG sequence for enzymatic modification were fused at either the extracellular N-terminus or the intracellular C-terminus. These plasmids were introduced into cells by retrovirus-mediated gene transfer. Similarly, Ba/F3 cells expressing a chimeric receptor of c-Mpl were prepared using retrovirus-mediated gene transfer, as previously described^20^.

Ba/F3 cells were cultured in RPMI-1640 medium supplemented with 10% fetal bovine serum and 1 ng/mL murine IL-3 at 37 °C under 5% CO_2_. HeLa cells were cultured in Dulbecco’s modified Eagle’s medium supplemented with 10% fetal bovine serum and 0.5% penicillin/streptomycin. Cells suspended in culture medium were collected by centrifugation (100 g, 3 min). The cells were washed with phosphate buffered saline without calcium and magnesium (PBS) twice by resuspension and centrifugation and, finally, were suspended in PBS to prepare cell suspensions at the desired concentrations.

### Cell membrane sheet formation

PEG–lipid coating on glass substrates was performed by an amine coupling reaction between the NHS ester of lipidation reagent **1** and the amine moieties on collagen coated surfaces. Slide glasses (from ibidi GmbH, Munich, Germany) were first washed with isopropanol and acetone in an ultrasonic bath and then incubated overnight at room temperature in a collagen solution (0.3 mg/ml, pH 3). After incubation, the slide glasses were washed with Milli-Q water (Nihon Millipore, Tokyo, Japan) more than 3 times. Next, 100 μM lipidation reagent **1** in PBS, including 1% dimethyl sulfoxide (DMSO), was put onto the collagen coated slide glasses. After incubation for 1 h at 37 °C, the slide glasses were washed with Milli-Q water more than 6 times and then air dried for subsequent use.

To prepare cell membrane sheets by microfluidic laminar flow, a microfluidic device immobilizing cells in the flow path was prepared by combining the PEG-lipid coated glass substrate with a sticky-slide (from ibidi GmbH, Munich, Germany) which had a microchannel. When the sticky-slide was fitted to the glass, the microchannel (with a width, length and height of 5 mm, 48.5 mm and 0.1 mm) was closed by the PEG–lipid coated surface of the glass, establishing a flow path with a PEG–lipid coated bottom surface. The combined sticky-slide and glass were fixed in place with a set of handmade stainless steel clips (Supplementary Fig. S2). After formation of the flow path, 100 μL of a cell suspension was poured into each micro flow path, followed by incubation for 10 min at room temperature to enable immobilization. Then, the flow path was connected to a syringe pump (Legato 100, KD Scientific, Holliston, MA, USA), and PBS was poured into the flow path at a variety of velocities, from 0.1 to 50 mL/min, for 1 min, to induce cell fracture. Reynolds number of this microchannel is around 300, in the laminar regime, even at the maximum 50 ml/min. This value was calculated using Equation 1 and 2.

1 $$Re=\frac{\rho u{D}_{H}}{v}$$

2 $${D}_{H}=\frac{4A}{P}$$

(*Re*: Reynolds number, *ρ*: density, *u*: velocity, *D*_*H*_: hydraulic diameter, *v*: dynamic viscosity, *A*: cross-sectional area, *P*: wetted perimeter of the cross-section)

The wall shear stress was calculated using Equation 3–5, which are adequate in case of Poiseuille flow.

3 $${\tau }_{W}=\mu \frac{du(y)}{dy}{| }_{y=0}$$

4 $$u(y)=4{u}_{max}\frac{y}{h}(1-\frac{y}{h})$$

5 $${u}_{max}=\frac{3}{2}\bar{u}$$

(${\tau }_{W}$: wall shear stress, *μ*: viscosity, *u*: velocity, *h*: height of flow channel, *u*_*max*_ = maximum velocity, $\bar{u}$: mean velocity).

As a control experiment, immobilized cells were also fractured by sonication, as described previously^10^. First, cells were immobilized on PEG–lipid modified glass substrates. The immobilized cells were then exposed to a hypotonic buffer (pH 7.2, 20 mM 4-(2-hydroxyethyl)-1-piperazineethanesulfonic acid (HEPES) supplemented with 120 mM monopotassium glutamate, 20 mM potassium acetate and 2 mM EGTA for 20 min, then fractured by brief sonication with a homogenizer (UD-200, Tomy tech., Tokyo, Japan).

### Confocal fluorescence imaging

Cells were fluorescently stained with DiI, CalceinAM, Alexa647–PEG-lipid, FL–PEG–lipid or Hoechst33342, using suitable methods for each reagent. DiI, CalceinAM and Hoechst33342 were each used according to the manufacturer’s protocol. Alexa647-PEG–lipid and FL-PEG–lipid were first dissolved in DMSO at 10 mM, and each dye solution was then diluted 1:1000 with PBS. This diluted solution was added to the cell pellets. After incubation in the dye solution at 37 °C for 15 min, cells were washed twice with PBS. For immunostaining of the cell membrane surface, anti–HA rabbit polyclonal antibody FITC conjugate was used. A 200 μL aliquot of antibody solution (1 μg/mL in PBS) was poured into the flow path after formation of the cell membrane sheets. After incubation for 30 min at room temperature, the membrane sheets in the flow path were washed with 300 μL PBS.

These multiple stained cells were observed by confocal fluorescence microscopy, before and after cell fracture. Confocal fluorescence images were obtained with a confocal laser-scanning microscope (LSM510, Carl Zeiss, Jena, Germany) equipped with 20× or 40× objective lenses. Image analysis was performed with the associated software (ImageJ software (NIH, Bethesda, MD). The structures with only red fluorescence were counted as the cell membrane sheet, and those with both red and green fluorescence were as the whole cell. The ratio of the membrane sheet formation was determined by dividing the number of the cell membrane sheets with the total number of the membrane sheets and the whole cells in each frame at nine different areas. The formation ratios were obtained from three independent trials and represented as means ± standard error (n = 18).

### Scanning electron microscopy (SEM) imaging

Cell membrane sheets from Ba/F3 cells were prepared as described above and chemically fixed with 2.5% glutaraldehyde in phosphate buffer (50 mM, pH 7.4) at room temperature for 1 h. After washing with Milli-Q water 3 times, membrane sheets were fixed further with 1% OsO4, in same buffer, at room temperature for 1 h. Then, the membrane sheets were washed with Milli-Q water 3 times, dehydrated in a graded series of ethanol-water solutions and dried by the critical point drying method with a Leica EM CPD 300 (Leica, Germany). The surface was coated with Pd-Pt (E-1030, Hitachi, Tokyo, Japan) for 2 min and then samples were observed with a scanning electron microscope (S-800, Hitachi, Tokyo, Japan).

### Fluorescence recovery after photo-bleaching (FRAP) analysis

FRAP experiments were performed under the same conditions as those for confocal fluorescence imaging. Cell membrane sheets were prepared from Ba/F3 cells stained with DiI. For bleaching, an He/Ne laser (543 nm) was used with the following conditions: bleaching iteration = 50, laser intensity = 200 and bleaching diameter = 2.3 μm. The fluorescence intensities of the bleached areas were tracked using the time-series mode of the operation software from 5 s before bleaching to 25 s after bleaching. Normalized intensity data were analyzed as previously described^12^. The half-times obtained were converted to 2-dimensional membrane diffusion constants, as previously described^35^. The time-course plots of the normalized fluorescence intensities obtained from the FRAP experiment are shown in Supplementary Fig. S6.

### Detection of receptor phosphorylation

An artificial chimeric receptor^19^, composed of anti-fluorescein (FL) single-chain Fv (ScFv) and thrombopoietin receptor (c-Mpl), was employed. This chimeric receptor (named ML0-type-c-Mpl) was reported to be phosphorylated in response to binding of FL modified bovine serum albumin (FL–BSA) to the extracellular anti-FL ScFv domain. Ba/F3 cells expressing the chimeric receptor were cultured in a depletion medium, without IL-3, at 37 °C for 12 h and then stimulated with 1 μg/mL FL–BSA at 37 °C for 15 min. Cells were then washed, resuspended in 2 mM Na_3_VO_4_ in PBS and poured into the flow path. Cell membrane sheets were prepared as described above and then stained with an anti-p-Tyr antibody AlexaFluor405 conjugate, after blocking with 1% BSA in PBS for 1 h. Samples were then analyzed by confocal microscopy as described above. Quantitative image analysis was performed as follow. First, the background was subtracted by the rolling ball method to cancel the reflection from the glass surface. Then, the membrane sheets of individual cells were defined from DiI-stained cell membrane images, and the Alexa Fluor 405-fluorescent intensity of the labeled antibody in each membrane sheet was quantified.

### Enzymatic modification of GPCR

SrtA was expressed and purified as previously described^26^. Fluorescently labeled peptide (GGGYC–Alexa488) was prepared by standard Fmoc solid-phase peptide synthesis. The cysteine residue of the GGGYC peptide was modified with Alexa Fluor 488-maleimide in 25 mM 3-morpholinopropane-1-sulfonic acid (MOPS) buffer (pH 6.8) for 6 h at room temperature. The product, GGGYC–Alexa488 was purified by reversed phase high performance liquid chromatography (RP-HPLC, Jasco, Tokyo, Japan) and identified by matrix-assisted laser desorption and ionization time-of-flight mass spectrometry, as previously described^36^.

Cell membrane sheets were prepared from Ba/F3 cells expressing G2A–HA as described above and then incubated at 37 °C for 1 h in reaction buffer (pH 8.0, 35 μM SrtA, 80 μM GGGYC–Alexa488, 50 mM Tris-HCl, 150 mM KCl and 10 mM CaCl_2_). After the reaction, the membrane sheets were washed with 300 μL PBS and analyzed by confocal microscopy as described above. Quantitative image analysis was performed as described above. The Alexa Fluor 488-fluorescent intensity of the modified receptor in each membrane sheet was quantified.

### Light–guided patterning of the cell membrane sheets

The photo–cleavable lipidation reagent **2**, based on PEG–lipid, was synthesized and used for light-guided patterning of the cell membrane sheets, as previously described^28^. The PEG–lipid coated surface was prepared essentially as described above. Next, a pattern of ultraviolet light (360 ± 5 nm) was irradiated through the photomask with an array of micro-sized dots (diameter, 10 μm). When a cell suspension was poured into the flow path, cells were selectively immobilized on the non-irradiated areas, resulting in preparation of a single cell array. Then, the immobilized cells were fractured as described above. The resulting array of cell membrane sheet was stained with FL–PEG–lipid and analyzed by confocal microscopy.

## Electronic supplementary material

Supplementary information
