# Supplementary material for: Microfluidic preparation of anchored cell membrane sheets for in vitro analyses and manipulation of the cytoplasmic face
Source: Sci Rep. 2017 Nov 2;7:14962. doi: 10.1038/s41598-017-14737-7 (PMC5668413; doi:10.1038/s41598-017-14737-7)
Supplement: Supplementary file 1 — Supplementary information [file 41598_2017_14737_MOESM1_ESM.pdf]

Supplementary Information

Microfluidic preparation of anchored cell membrane sheets for *in vitro* analyses and manipulation of the cytoplasmic face

Shin Izuta[1], Satoshi Yamaguchi[2,3]*, Ryuji Misawa[1], Shinya Yamahira[1], Modong Tan[1], Masahiro Kawahara[1], Tomoko Suzuki[4], Tomoko Takagi[4], Kae Sato[4], Motonao Nakamura[5], Teruyuki Nagamune[1] & Akimitsu Okamoto[1,2]*

[1]*Department of Chemistry and Biotechnology, The University of Tokyo, 7-3-1 Hongo, Bunkyo-ku, Tokyo 113-8656, Japan.*
[2]*Research Center for Advanced Science and Technology, The University of Tokyo, 4-6-1 Komaba, Meguro–ku, Tokyo 153-8904, Japan*
[3]*PRESTO, Japan Science and Technology Agency (JST), Tokyo, Japan*
[4]*Department of Chemical and Biological Sciences, Japan Women's University, 2-8-1 Mejirodai, Bunkyo-ku, Tokyo 112-8681, Japan*
[5]*Department of Life Science, Faculty of Science, Okayama University of Science, 1-1 Ridai-cho, Kita-ku, Okayama-shi, Okayama 700-0005, Japan*

*Corresponding authors: Satoshi Yamaguchi, Research Center for Advanced Science and Technology, The University of Tokyo, 4-6-1 Komaba, Meguro-ku, Tokyo 153-8904, Japan.
E–mail: yamaguchi@bioorg.rcast.u-tokyo.ac.jp

Akimitsu Okamoto, Department of Chemistry and Biotechnology, The University of Tokyo, 7-3-1 Hongo, Bunkyo-ku, Tokyo 113-8656, Japan.
Tel: +81-3-5452-5200
Fax: +81-3-5452-5209
E–mail: okamoto@chembio.t.u-tokyo.ac.jp

1. Fluorescently labeled PEG–lipids

Fluorescently labeled PEG–lipids (Fig. S1) were synthesized essentially as previously reported[S1]. *N*-boc-amine-PEG–NHS was lipidated with DOPE using the amine coupling reaction and, after deprotection, FITC and Alexa647–NHS were reacted with the amine moiety of the PEG–lipid.

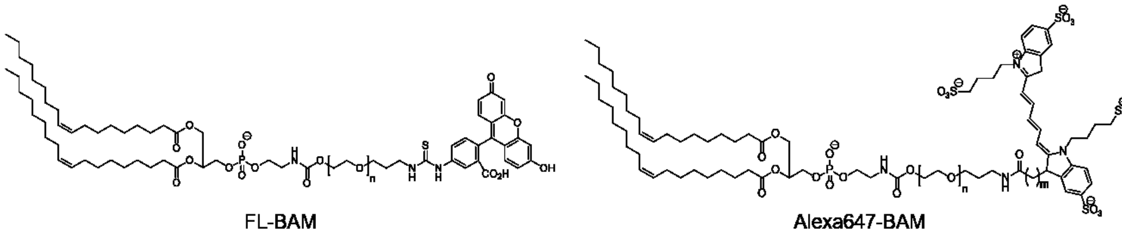

Figure S1. Chemical structures of reagents used for membrane staining.

2. Detailed protocols of microfluidic preparation of cell membrane sheets

To prepare cell membrane sheets by microfluidic laminar flow, a microfluidic device immobilizing cells in the flow path was prepared by combining the PEG-lipid coated glass substrate with a sticky-slide (catalog number: 81128, ibidi GmbH, Munich, Germany) which had a microchannel. The microchannel had a width, length and height of 5, 48.5 and 0.1 mm, respectively. When the sticky-slide was fitted to the glass, the microchannel was closed by the PEG–lipid coated surface of the glass, establishing a flow path with a PEG–lipid coated bottom surface. The glue on the surface of this sticky-slide was removed and the slide incubated in 1% BSA (Sigma Aldrich) for 10 min. After washing the sticky-slide with MilliQ water, both the sticky-slide and the glass were fitted together in PBS to prevent trapping of air in the flow path. The combined sticky-slide and glass were fixed in place with a set of handmade stainless steel clips (Fig. S2). After formation of the flow path, 100 μL of a cell suspension was poured into each micro flow path, followed by incubation for more than 10 min at room temperature to enable immobilization. Then, the flow path was connected to a syringe pump (Legato 100, KD Scientific, Holliston, MA, USA) with tubes (LMT-55, Saint-Gobain, Courbevoie, France) and connectors (VRF106 and VFT106, ISIS Co., Ltd., Osaka, Japan). PBS was poured into the flow path at a variety of velocities, from 0.1 to 50 mL/min, for 1 min, to induce cell fracture.

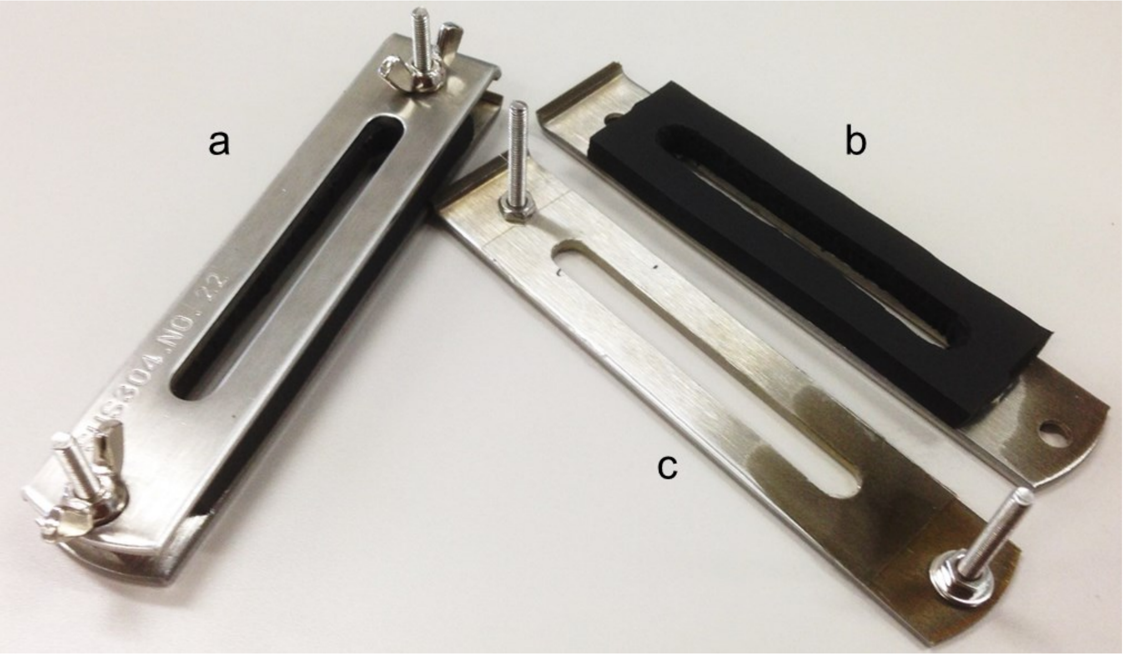

Figure S2. Photograph of handmade metal clips for flow path formation. a) combined clips; b) the upper part of the clip, which contacts the sticky-slide in the flow path; c) the bottom part of the clip, which contacts the slide glass.

3. Confocal fluorescence imaging of the cell membrane sheets of He/La cells

He/La cells were harvested and immobilized on the PEG–lipid surface, and after incubation for 10 min, similar to Ba/F3 cells, the cell membrane sheets were obtained by the present microfluidic cell fracture (Fig. S3a,c). In the case of adherent cells, it is previously reported that the immobilized cells adhere and expand on the PEG-lipid-modified collagen surface after long incubation under physiological conditions[S2]. In this study, He/La cells were also confirmed to expand on the surface after incubation for more than 1 hour at 37 °C. After adhesion and expansion, the efficiency of the cell membrane sheet formation from the well-spread cells was observed to decrease (Fig. S3b,c). The reason of this decrease can be explained in accordance with decrease in cell height and formation to the streamline form of the spread cells. These changes in cell shape are assumed to lead to reduction of shear stress on the cells.

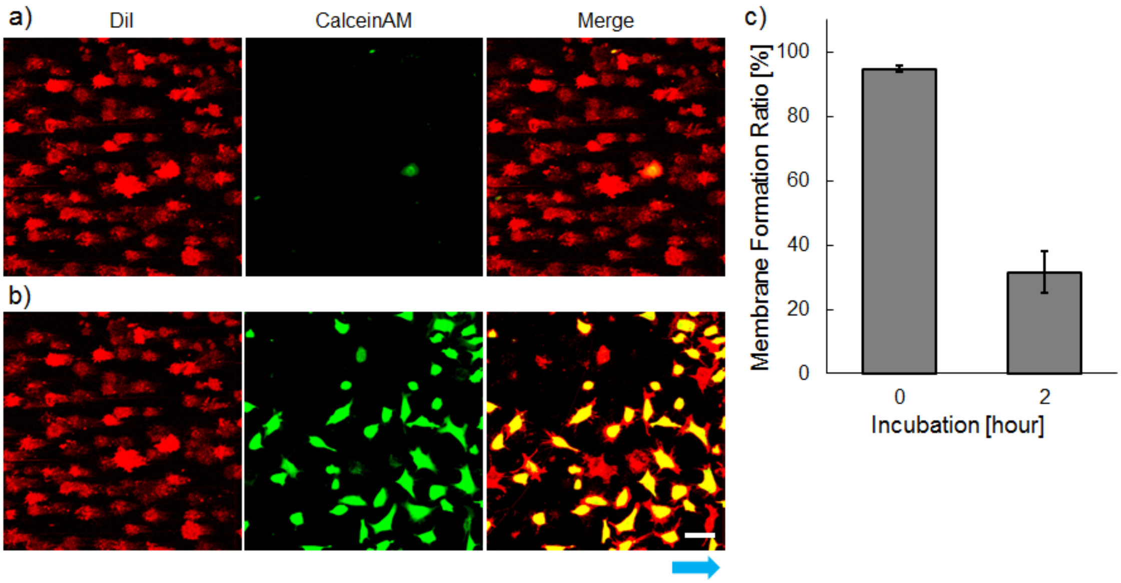

Figure S3. Confocal microscopic images of He/La cells after microfluidic cell fracture and the ratio of the cell membrane sheet formation. a,b) Fluorescent images of DiI-stained plasma membrane (red, *left*) and CalceinAM-stained cytoplasm (green, *center*) and their merged image (*right*) after microfluidic treatment (50 mL/min × 1 min) were obtained from He/La cells which were incubated on the PEG–lipid surface for a) 10 min and b) 2 hours. The 2 hour-incubation was done in DMEM (supplemented with 10% FBS) at 37 °C under 5% CO_2, to make HeLa cells extended on PEG-Lipid surface. Scale bar, 50 µm. Blue arrow shows the direction of flow for cell fracture. c) The ratio of the cell membrane sheet formation (n = 16). Values are means ± standard error.

4. Immunostaining of whole cells expressing the antigen on cell surfaces

In addition to the samples shown in Fig. 3, whole cells expressing HA–G2A, not exposed to microfluidic laminar flow, were also observed after immunostaining (Fig. S4). In these samples, on the bottom plasma membrane of the immobilized cells, the HA-tag on the extracellular domain of G2A did not stain with anti-HA antibody FITC conjugate, whereas it was clearly stained on the top and side plasma membrane. These results strongly suggested that the anti-HA antibody FITC conjugate could not access the space between the membrane sheet and the glass substrate.

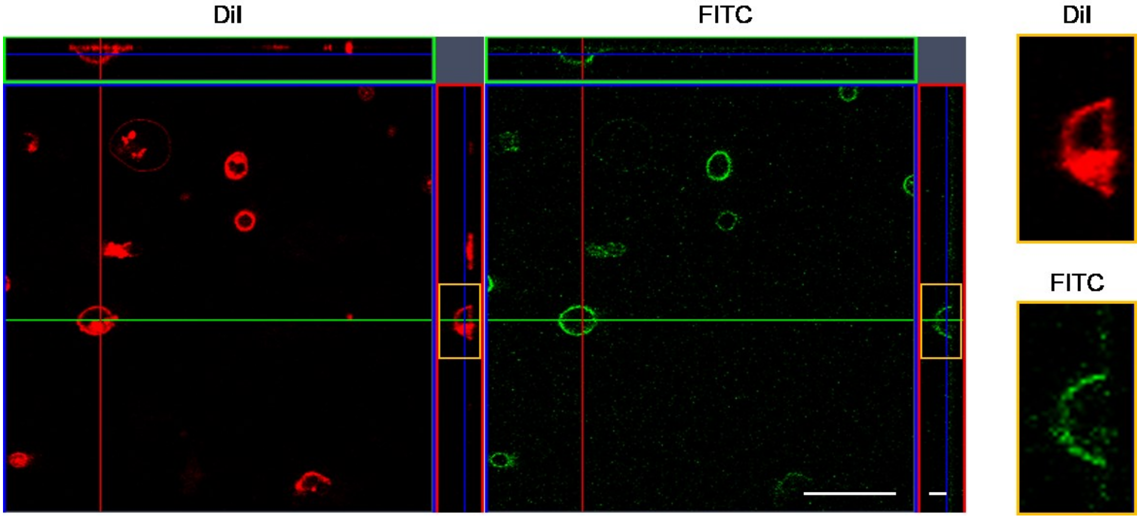

Figure S4. Cross sections of the confocal 3D reconstruction images of whole Ba/F3 cells expressing HA-G2A, after immunostaining. The (left) x-y and (right top and bottom) x-z sections were obtained from the 3D image, which was reconstructed by composing a series of 2D z-stack confocal images of cells stained with DiI (plasma membrane, red) and anti-HA antibody FITC conjugate (HA-tag, green). In this case, DiI-staining and immunostaining were performed before and after cell immobilization, respectively. The x–z section images of (right top) DiI and (right bottom) FITC fluorescence were taken at the green line in the x-y section images. In the x-z FITC image, the bottom membrane of the immobilized cell was clearly not stained. Scale bar, 50 μm (width), 10 μm (height).

5. Scanning electron microscopy (SEM) imaging

In addition to those shown in Figs. 4, SEM images of a whole cell and membrane sheets are shown in Fig. S5. Traces were observed behind the fractured membrane sheets in Fig. 4a and Fig. S5b. Therefore, the enlarged images of the traces in Fig. 4a were also obtained (Fig. S6).

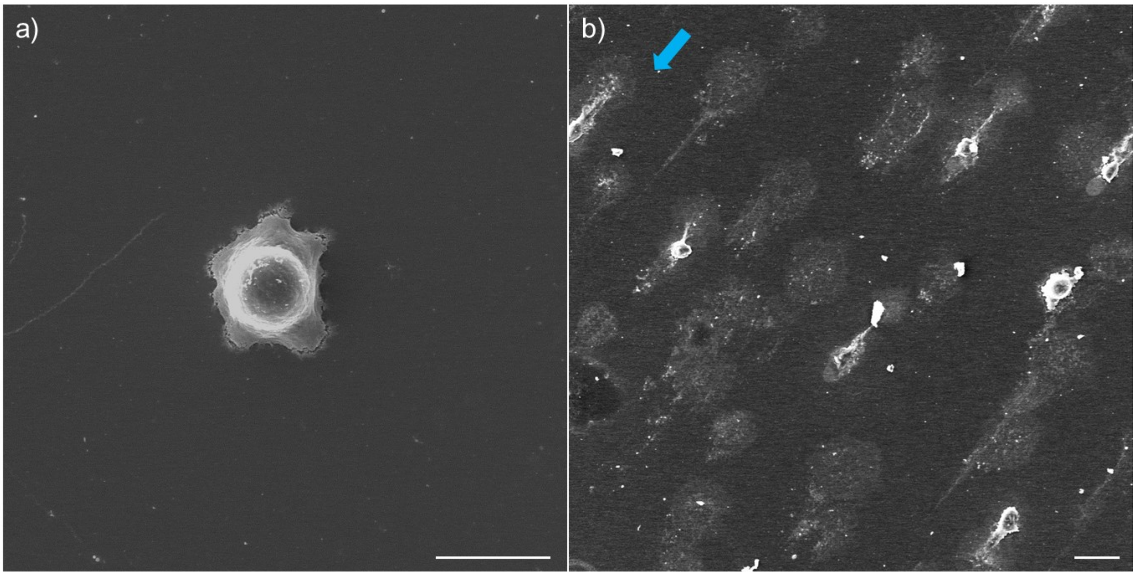

Figure S5. SEM images of (a) a whole cell and (b) cell membrane sheets formed by the microfluidic method. Scale bars, (a) 10 μm, (b) 20 μm. Blue arrow shows the direction of flow for cell fracture.

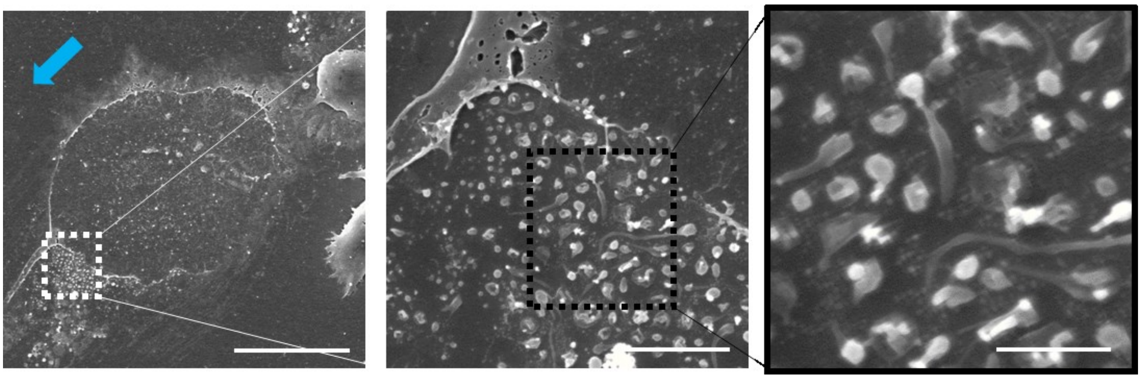

Figure S6. SEM images of traces behind fractured membrane sheet. Scale bars, 15 μm (*left*), 2.7 μm (*center*) and 1.0 μm (*right*), respectively. The arrow indicates the direction of the flow. Blue arrow shows the direction of flow for cell fracture.

6. Fluorescence recovery after photo-bleaching (FRAP) analysis

The time-course plots of the normalized fluorescence intensities obtained from the FRAP experiment are shown in Fig. S7.

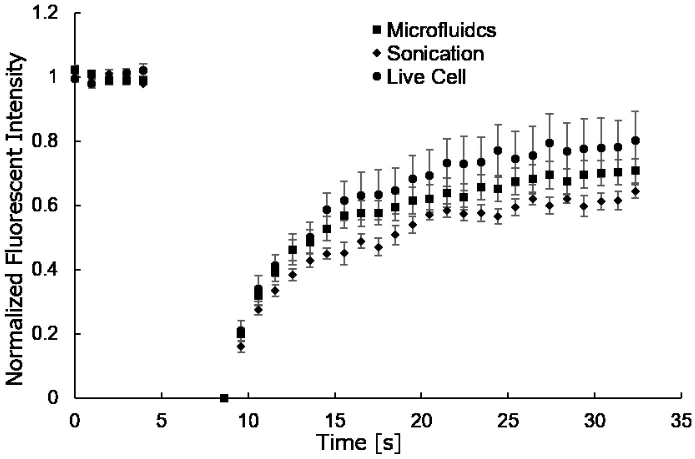

Figure S7. Time-course plots of the normalized fluorescence intensities in FRAP experiments on intact living cells and cell membrane sheets. FRAP data were obtained with the bottom membrane of whole Ba/F3 cells stained with DiI (closed circles), cell membrane sheets formed from these cells with the microfluidic method (closed squares) and with the sonication method (closed diamonds). Raw data were normalized to intensities obtained just before bleaching. Values are means ± standard deviation (n = 10).

References

S1) Kato, K., Itoh, C., Yasukouchi, T. & Nagamune, T., Rapid protein anchoring into the membranes of mammalian cells using oleyl chain and poly(ethylene glycol) derivatives. *Biotechnol. Prog.* **20**, 897–904 (2004).

S2) Yamahira, S., Yamaguchi, S., Kawahara, M. & Nagamune, T., Collagen surfaces modified with photo-cleavable polyethylene glycol-lipid support versatile single-cell arrays of both non-adherent and adherent cells. *Macromol. Biosci.* **14**, 1670-1676 (2014).
